# Postoperative hysterical symptoms in a patient with epidural catheter

**DOI:** 10.4103/0019-5049.68390

**Published:** 2010

**Authors:** M Ravi, PB Ramesh Kumar, K Dinesh, VD Deepak

**Affiliations:** Department of Anaesthesia and Intensive Care, Sri Devaraj Urs Medical College, Tamaka, Kolar, Karnataka, India

Sir,

We had a patient in the postoperative ward presenting with irritability, restlessness, disorientation, shouting irrelevant words, and was haemodynamically stable and saturation was normal. This was 30 minutes after total abdominal hysterectomy done under combined spinal epidural anaesthesia (Epidural placement confirmed by activating in the theatre).

On evaluation we came to know that patient had complained of pain and a trained nurse had given a top up of 0.125% Bupivacaine 6 ml through the Epidural catheter after negative aspiration of CSF/ Blood. After this immediately patient started behaving with the above symptoms and complaining of pain. She was hemodymically stable. She was not responding to oral commands, thrashing her hands and legs on the bed, was given Injection Tramadol 50 milli gram intravenously, but still the symptoms persisted, Injection Haloperidol 1milli gram was given intravenously and after 5 minutes she was calm and slept off.

We removed the epidural catheter as she was moving/thrashing on the bed earlier, the tip was blood stained (may be due to the movement/intravascular migration).[1] After two hours, the patient was normal and did not remember any of the events except of complaining of pain in the post operative period.

We observed that she was anxious prior to the procedure and no history of hysterical behaviour in the past was observed.[2] Anxiety and pain would have caused this behaviour. Intravascular migration of catheter or displacement may also contribute in terms of pain because of improper drug delivery. Proper counselling and patient preparation/education matters in perioperative patient care.
